# Protective Effects of Bacteriophages against *Aeromonas hydrophila* Causing Motile Aeromonas Septicemia (MAS) in Striped Catfish

**DOI:** 10.3390/antibiotics7010016

**Published:** 2018-02-25

**Authors:** Tuan Son Le, Thi Hien Nguyen, Hong Phuong Vo, Van Cuong Doan, Hong Loc Nguyen, Minh Trung Tran, Trong Tuan Tran, Paul C. Southgate, D. İpek Kurtböke

**Affiliations:** 1 GeneCology Research Centre, Faculty of Science, Health, Education and Engineering, University of the Sunshine Coast, 90 Sippy Downs Drive, Sippy Downs, QLD 4556, Australia; tuan.son.le@research.usc.edu.au; 2 Research Institute for Marine Fisheries, 224 Le Lai, Ngo Quyen, Hai Phong 180000, Vietnam; 3 Research Institute for Aquaculture No. 2, 116 Nguyen Dinh Chieu, District 1, Ho Chi Minh 700000, Vietnam; nguyenhien05@gmail.com (T.H.N.); vohongphuong@gmail.com (H.P.V.); vancuongdisaqua@gmail.com (V.C.D.); hongloc@gmail.com (H.L.N.); trung16893@yahoo.com.vn (M.T.T.); tuantran_695@yahoo.com.vn (T.T.T.); 4 Australian Centre for Pacific Islands Research and Faculty of Science, Health, Education and Engineering, University of the Sunshine Coast, Maroochydore, QLD 4556, Australia; psouthgate@usc.edu.au

**Keywords:** *Aeromonas hydrophila*, Motile Aeromonas Septicemia, MAS, multiple-antibiotic-resistance, bacteriophage, biological control, striped catfish (*Pangasianodon hypophthalmus*)

## Abstract

To determine the effectivity of bacteriophages in controlling the mass mortality of striped catfish (*Pangasianodon hypophthalmus*) due to infections caused by *Aeromonas* spp. in Vietnamese fish farms, bacteriophages against pathogenic *Aeromonas hydrophila* were isolated. *A. hydrophila*-phage 2 and *A. hydrophila*-phage 5 were successfully isolated from water samples from the Saigon River of Ho Chi Minh City, Vietnam. These phages, belonging to the *Myoviridae* family, were found to have broad activity spectra, even against the tested multiple-antibiotic-resistant *Aeromonas* isolates. The latent periods and burst size of phage 2 were 10 min and 213 PFU per infected host cell, respectively. The bacteriophages proved to be effective in inhibiting the growth of the *Aeromonas* spp. under laboratory conditions. Phage treatments applied to the pathogenic strains during infestation of catfish resulted in a significant improvement in the survival rates of the tested fishes, with up to 100% survival with MOI 100, compared to 18.3% survival observed in control experiments. These findings illustrate the potential for using phages as an effective bio-treatment method to control Motile Aeromonas Septicemia (MAS) in fish farms. This study provides further evidence towards the use of bacteriophages to effectively control disease in aquaculture operations.

## 1. Introduction

Striped catfish (*Pangasianodon hypophthalmus*) is one of the most important farmed fish species, especially in Vietnam, Thailand, Cambodia, Laos and, more recently, the Philippines and Indonesia [1]. Vietnam supplied 90% of catfish production with a value of US$1.1 to 1.7 billion in 2015. Motile Aeromonas Septicemia (MAS), also called haemorrhage disease or red spot disease, causes great losses for farmers (up to 80% mortality) and presents in fish with clinical signs of haemorrhages on the head, mouth, and at the base of fins, a red, swollen vent, and the presence of pink to yellow ascitic fluid [2]. *Aeromonas hydrophila*, *Aeromonas caviae*, and *Aeromonas sobria* species were often isolated from diseased catfish, and new species such as *Aeromonas dhakensis* and *Aeromonas veronii* were also reported by using molecular methods based on the sequencing of the *rpo*D gene [3].

Multiple antibiotic resistance (MAR) of *A. hydrophila* strains has been reported in different countries. Vivekanandhan et al. [4] tested 319 strains of *A. hydrophila* isolated from fish and prawns in South India and indicated that all of them were resistant to methicillin, rifampicin, bacitracin, and novobiocin (99%). Moreover, 21 *Aeromonas* spp. isolated from carp showed resistance to ampicillin and penicillin [5]. Recently, Thi et al. [6] tested antibiotic resistance of 30 strains of *A. hydrophila* isolated from diseased striped catfish in the Mekong Delta from January 2013 to March 2014. The study found that *A. hydrophila* isolates were highly resistant to tetracycline and florfenicol and were completely resistant to trimethoprim, sulfamethoxazole, ampicillin, amoxicillin, and cefalexine.

ALPHA JECT ^®^ Panga 2 vaccine, protecting against *Edwardsiella ictaluri* and *A. hydrophila*, has been approved for market in Vietnam since the early 2017 (https://www.pharmaq.no/updates/pharmaq-fish-va/). However, the cost-effectiveness of vaccine use in catfish production is another obstacle in intensive catfish production. Moreover, the development of a commercial vaccine against *A. hydrophila* has been slow because *A. hydrophia* is biochemically and serologically heterogeneous [7]. Therefore, there is a need for effective, environmentally safe control measures for managing MAS in catfish. 

One approach has been the use of bacteriophages (phages) to control pathogenic bacteria in aquaculture operations. Recently, studies related to the use of phages specific to *A. hydrophila* in aquaculture have gained attention. Hsu et al. [8] isolated two *A. hydrophila* phages and three *Edwardsiella tarda* phages to treat disease in eels (*Anguilla japonica*) in vitro. The phages reduced bacterial density by about 1000 times after 2 h when the MOI was 11.5 at 25 °C in the fluid environment. El-Araby et al. [9] demonstrated the effectiveness of bacteriophage ZH1 and ZH2 treatment against *A. hydrophila* in Tilapia, improving the survival rates by up to 82%.

However, so far, treatments using bacteriophages against pathogens causing MAS in catfish have not been studied extensively. The objective of this study was, therefore, to isolate bacteriophages infective in pathogenic *A. hydrophila* with a long-term objective to eradicate this disease-causing pathogen in aquaculture operations.

## 2. Results

### 2.1. Prophage Induction

No reduction in the optical density of bacterial suspension treated with Mitomycin C (Table S1, Supplementary Materials) and no clear zones from the spot technique were observed. Therefore, it was concluded that there was no prophage in *A. hydrophila* N17.

### 2.2. Antibiotic Susceptibility

All isolates were completely (100%) resistant to oxytetracycline, ampicillin, gentamycin and amoxicillin/clavulanic acid, enrofloxacin, and bactrim. Nearly all isolates (83.3%) were resistant to kanamycin and 33.3% were resistant to tetracycline, doxycycline, and ciprofloxacin (Table 1).

### 2.3. Isolation and Characterization of Bacteriophages

**Figure 1:**
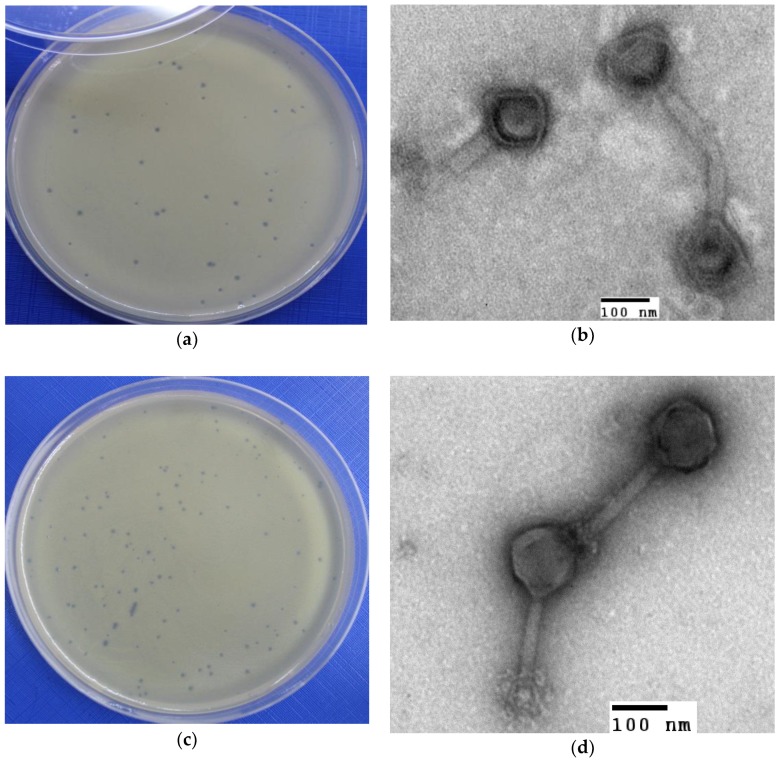
Plaque formation and microphotograph of *A. hydrophila* phages. (**a**,**b**) Φ2 and (**c**,**d**) phage Φ5.

The A. hydrophila-phage 2 (or Φ2) and A. hydrophila-phage 5 (or Φ5) were successfully isolated against the propagation hosts used (Figure 1 and Table 2).

Φ2 had an isometric head of 129 nm in diameter with a tail sheath 173 nm long and 15 nm wide. Φ5 was composed of: (i) an isometric head of 120 nm in diameter, (ii) a tail sheath of 198 nm in length and 15 nm in width. All of the phages had contractile tails (Figure 1 and Table 2).

Both phages produced clear plaques with diameters of 0.1 mm (Figure 1).

**Figure 2:**
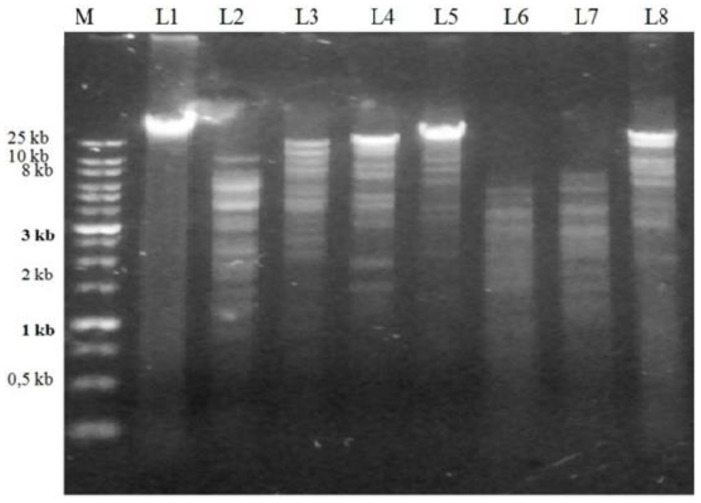
Restriction enzyme-digested fragments of the genomic DNA of *A. hydrophila*-phage 2. Footnote: Lane M: 1kb Plus Opti-DNA Marker (ABM, Canada); Lane L1: genomic DNA of Φ2; Lanes L2–L8: genomic DNA of Φ2 digested with EcoRV; EcoRI; Ncol; SalI; MspI; XmnI; KpnI, restriction enzymes respectively.

The genome size of the phage isolates was above 20 kb. The genomic material of the isolated phages was not digested by Mung bean nuclease and RNase A. Since Mung bean nuclease specifically cuts single-stranded nucleic acids of both DNA and RNA, it was concluded that the genomic DNA of both phages was double-stranded. RNA nucleic acids are degraded by RNase A, therefore, the nucleic acids of Φ2 and Φ5 were determined as double-stranded DNA (dsDNA) (Figure 2). The phages Φ2 and Φ5 belong to the Myoviridae family.

### 2.4. Host Range

Phage 2 and phage 5 were found to inhibit the growth of all *A. hydrophila* strains tested. None of the other 27 species was found to be susceptible to these phages (Table S2, Supplementary Materials).

### 2.5. Adsorption Rate of Phages and One-Step Growth Curve

**Figure 3:**
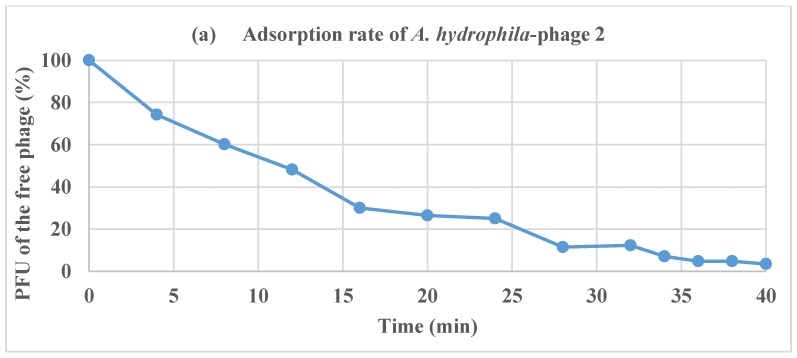

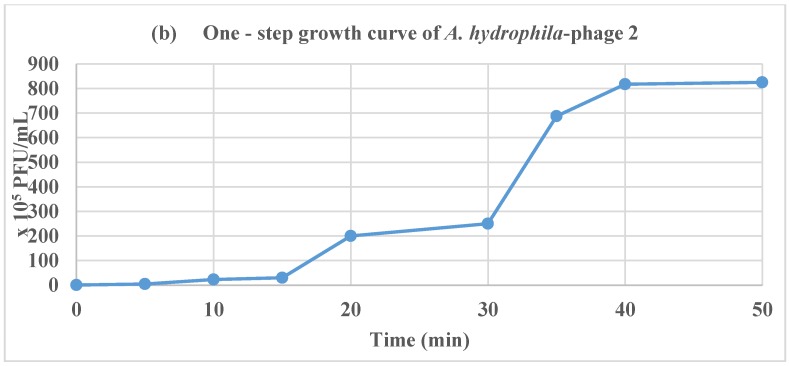
(**a**) Adsorption rate and (**b**) one-step growth curves of Φ2.

The number of free phages in suspension decreased over time, as illustrated in the adsorption curve (Figure 3a). At 40 min, the percentage of Φ2-infected bacteria was over 90%.

The one-step growth experiment (Figure 3b) results revealed that the latent period and burst size of Φ2 were 10 and 213 PFU per infected host cell, respectively.

### 2.6. Inactivation of Aeromonas Species in Vitro

**Figure 4:**
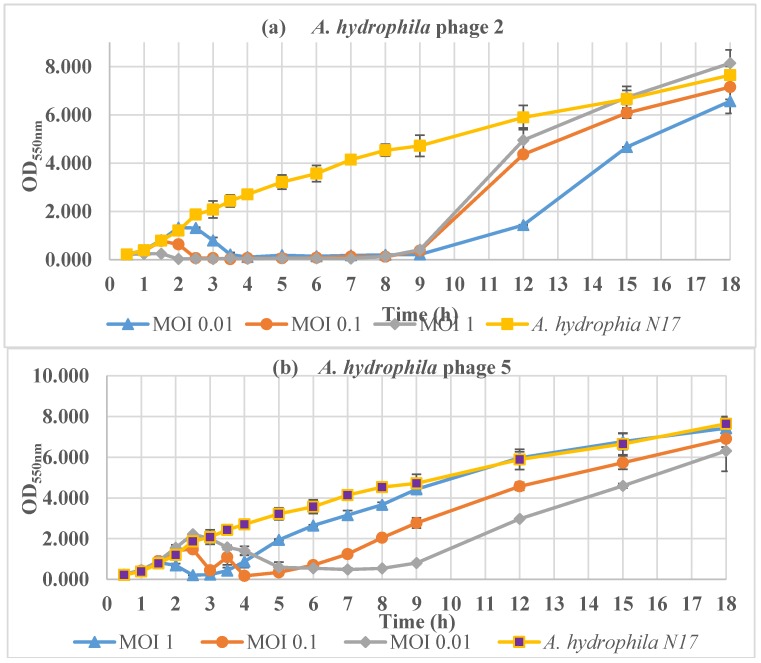
Inactivation of *A. hydrophila* N17 by the phages (**a**) Φ2 and (**b**) Φ5 at different MOI (0.01, 0.1 and 1).

The bacterial concentration (OD_550nm_ values) of the uninfected control (only *A. hydrophila* N17) increased continuously during 18 h of incubation. In contrast, during the infection with Φ2 at MOI 1, MOI 0.1, and MOI 0.01 bacterial growth began to be inhibited at 1, 2, and 2.5 h, respectively, and the inhibition was maintained up to 8 h (Figure 4a). Then, the bacterial concentration increased as a consequence of the development of phage-resistant *A. hydrophila* cells.

The lowest OD_550nm_ value was 0.177 ± 0.023 after 4 h of incubation of Φ5 at MOI 0.1. There was a significant decline in the bacterial concentration (MOI 0.01, 0.1, and 1) in the first 3 h, followed by low level stabilization in the next 1, 2, and 4 h for MOI 1, 0.1, and 0.01, respectively (Figure 4b). Then, the bacterial concentration underwent a turnaround because of the development of phage-resistant *A. hydrophila* cells.

### 2.7. Phage Treatment of Infected Fish

**Figure 5:**
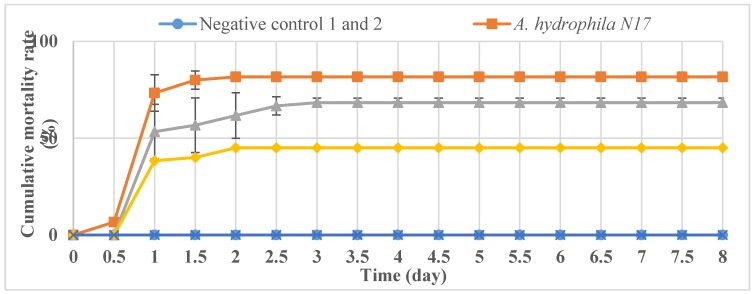
Cumulative mortality rates (%) of striped catfishes obtained in challenging experiments using *A. hydrophia* N17 and the phage cocktail at the different MOIs (0.01, 0.1, and 1). The ratio of Φ2 to Φ5 in a phage cocktail was 1:1.

The negative control 1 (fishes with no injection) and negative control 2 (fishes injected with the growth medium filtered to remove bacterial cells) showed no mortality of catfish (Figure 5), indicating that the uninfected, control medium did not have any detrimental effect on fish health.

Catfish in the positive control groups (infected with *A. hydrophila* N17) that were not treated with bacteriophages started to die at a constant rate starting from post-infection day two, with a cumulative mortality rate of 81.67 ± 2.36% (Figure 5).

In contrast, the fish treated with the phages showed lower mortality rates at each different MOI (*p* < 0.01). While no mortality was observed in the groups treated with MOI 100, the cumulative mortalities in the other groups were 45% (MOI 1) and 68.33 ± 2.36% (MOI 0.01) at the end of the eight-day experiment (Figure 5).

## 3. Discussion

The findings of this study demonstrate that the examined *Aeromonas* spp. were resistant to multiple antibiotics and were thus able to cause high mortality rates in catfish in Vietnam, in spite of the use of various antibiotic treatments. In the bacteriophage treatments, however, Φ2 and Φ5 were able to lyse all tested *A. hydrophila* strains, displaying strong inhibition also of the virulent *A. hydrophila* strains carrying many virulence genes. Therefore, Φ2 and Φ5 are promising candidates for the application of a phage therapy to control *Aeromonas* infection in catfish.

Phage Φ2 and Φ5 were found to belong to the *Myoviridae* family, and our findings are in line with those of Ackermann [10] who indicated that 33 of a total of 43 *Aeromonas* phages he investigated were tailed and belonged to the *Myoviridae* family. Recently, other *Aeromonas* phage studies against different *Aeromonas* species by Haq et al. [11], Jun et al. [12], and Kim et al. [13] also reported that all phages they identified belonged to the *Myoviridae* family. Therefore, *Myoviridae* family members are most likely to be abundant in natural environments.

There was a correlation between the diameter of the plaques observed and the latent period and burst size for the *A. hydrophila* phage. The Φ2 had a short latent period (10 min), and these findings are in line with another study conducted by Anand et al. [14] who found that *Aeromonas* phage BPA 6 had a latent period of 10 min and a burst size of 244 PFU/cell.

The different MOI of Φ2 and Φ5 caused different bacterial growth patterns. The higher the MOI value, the sooner phage-resistant bacterial cells appeared. A similar result was noted by Kim et al. [13] for the phage PAS 1 against an *Aeromonas salmonicida* strain, indicating that bacterial resistance appeared after 3, 6, and 24 h at MOIs 10, 1, and 0.1, respectively.

Several *Aeromonas* phages, such as Aeh1, Aeh2, AH1 have also been reported [12,15,16]. However, there have been few reports demonstrating the successful use of phages for the treatment of *Aeromonas* infections in catfish. The treatment of catfish by an intraperitoneal (IP) injection illustrated significant protective effects, which increased the relative percentages of the survival rates observed for fish compared to the controls when the MOI increased. Our study revealed that in the MOI-100 experiment the relative percentage survival was 100%. The study of Jun et al. [12] showed that the relative percentage survival of fish treated with *A. hydrophila* phages pAh6-C and pAh1-C was 16.67 ± 3.82% and 43.33 ± 2.89%, respectively, when the fish were injected with the bacterium (2.6 × 10^7^ CFU/fish). However, the labour-intensive and time-consuming mode of delivery of bacteriophages can constitute a disadvantage for the treatment of fish by IP injection in catfish farms. Therefore, further studies should be conducted into whether phage treatments are effective when an on-farm oral method of administration is evaluated. With the use of bioreactors, large volumes of bacteriophages can be produced for bacteriophage incorporation into fish feed. Moreover, the survival of phages and their persistent survival on or in fish, as well as in phage-coated feed preparations should be studied under different environmental factors (e.g., temperature, salt concentration) to determine whether phages are able to persist and effectively reduce *Aeromonas* spp. levels in fish farms. In conclusion, this study demonstrates that phage treatment of *Aeromonas* spp. might be an effective tool to improve the survival of farmed catfish affected by MAS.

## 4. Materials and Methods

### 4.1. Aeromonas Species

Bacterial isolates stored at the Research Institute for Aquaculture No. 2 (Ho Chi Minh City, Vietnam) and the ATCC type strains of the pathogens are listed in Table S2. Isolates were previously obtained from diseased catfish in farms in the south of Vietnam (Table S2).

### 4.2. Prophage Induction

In order to choose an *Aeromonas* species as a propagation host for phage isolation, *A. hydrophila* N17 was subjected to a prophage induction test. The *Aeromonas* species was cultured in 10 mL fresh Luria-Bertani (or LB) broth (Sigma-Aldrich, St. Louis, MO, USA) and incubated at 30 °C on an orbital shaker operating at 150 rpm until reaching an OD_550nm_ of 0.2. Mitomycin C (Sigma-Aldrich) was added to a final concentration of 1 µg/mL and 5 µg/mL, and again the bacterial suspension was incubated at 30 °C on an orbital shaker operating at 150 rpm. The cell density of the bacteria (OD_550nm_) was monitored every 1 h for a 6 h period. At the end of the incubation, the bacterial suspension was centrifuged at 10,000 g for 15 min and filtered through a nitrocellulose filter (0.45 µm, Merck Millipore, Burlington, MA, USA) before spotting the filtrate onto an agar plate seeded with the host bacterium to confirm the presence of viable phage particles. A significant decrease in the cell density (OD_550nm_) suggested that prophages were released [17,18].

### 4.3. Antibiotic Susceptibility 

Antibiotic susceptibility tests of six *A. hydrophila* strains [3] were conducted against 10 different antimicrobial susceptibility discs (OXOID, Hampshire, UK) by the method recommended by the Clinical and Laboratory Standards Institute [19]. The antimicrobial agents tested included tetracycline (30 µg), doxycycline (30 µg), oxytetracycline (30 µg), bactrim (SMX/TMP) (23.75/1.25 µg), gentamycin (40 µg), kanamycin (30 µg), ciprofloxacin (10 µg), enrofloxacin (10 µg), ampicillin (33 µg), and amoxicillin-clavulanic acid (20/10 µg). 

The antimicrobial susceptibility of *Aeromonas* species is usually recorded using Enterobacteriaceae breakpoints [20]. Susceptible (S), intermediate resistance (I), and resistant (R) were evaluated according the criteria given in the Performance Standards for Antimicrobial Susceptibility Testing M100-S21 (2017, Table 2A-1, pages 33–39) [19]. Multi-antibiotic resistance (MAR) was recorded when the bacteria resisted to three or more antibiotics [21].

### 4.4. Isolation and Characterization of Bacteriophages

Phages were isolated from water samples from the Saigon River in the south of Vietnam against *A. hydrophila* N17 and they were purified following the methods described by Jun et al. [12].

Phage titres were determined using both surface spread [22,23] and double-layer [24] agar plaque assay techniques where agar plates were previously seeded with the *Aeromonas* sp. (×10^6^ CFU/mL).

For transmission electron microscopy (TEM): A 200 mesh copper grid was immersed in 40 µL of phage solution for five min before fixing the phage with glutaraldehyde solution (1%) for five min. Then, the phage samples were negatively stained with 5% (*w/v*) uranyl acetate and observed by TEM (JEOL JEM-1010) operating at a voltage of 80 kV at the Vietnam National Institute of Hygiene and Epidemiology. The phage morphology was determined using the criteria of the International Committee on Taxonomy of Viruses (ICTV) (http://www.ictvonline.org/) and Ackermann et al. [25].

Phage genomic DNA extraction and restriction analyses: Phage genomic DNA was extracted using the Phage DNA Isolation Kit (Norgen Biotek Corp, Thorold, Canada). The nature of the nucleic acids was determined by digestion with Mung bean nuclease and RNase A (ThermoFisher Scientific, Waltham, MA, USA) as per the manufacturer’s protocols. The genomic DNA phages were digested using the restriction enzymes: EcoRV, EcoRI, Ncol, SalI, MspI, XmnI, and KpnI, as per the manufacturer’s instruction (ThermoFisher Scientific). The DNA fragments were then electrophoresed at 120 V for 40 min.

### 4.5. Host Range

The method was adapted from Le et al. [23] and Goodridge et al. [26] with some modifications described below. The *Aeromonas* spp. (Table S2) were incubated overnight. Then, a 100 µL aliquot of each *Aeromonas* spp. culture (optical density of 0.5 at 550 nm) was spread on brain heart infusion agar (BHIA) (OXOID, UK) and dried for 20 min in a biological safety cabinet Class II. The host range of the phage was determined by pipetting 10 µL of phage preparation (~10^8^ PFU/mL) on lawn cultures of the strains. The plates were observed for the appearance of clear zones after incubation at 30 °C after 18 h.

### 4.6. Adsorption Rate of Phages 

Phage adsorption was studied using the method described previously [27]. A phage solution was added to 100 mL of log-phase growing *Aeromonas hydrophila* N17 culture (×10^7^ CFU/mL) in LB broth to get a final MOI of 0.1. The mixture was incubated at 30 °C. An aliquot of 1 mL was collected from the sample every two min over a period of 60 min. The sample was then centrifuged at 4000 g for 15 min, and then the supernatant was diluted with SM buffer + 1% chloroform (http://cshprotocols.cshlp.org/content/2006/1/pdb.rec8111.full?text_only=true). Then, the titers of unabsorbed free phages in the supernatant were determined by the double-layer agar technique, and the results were recorded as percentages of the initial phage counts. The percentages of free phages and the adsorption rates were calculated following the formula of Haq et al. [11].

### 4.7. One-Step Growth Curve

The phage and bacteria were prepared in the same way as in the adsorption method described above. At 40 min, when the adsorption rate was maximal, the mixture was further incubated at 30 °C with 150 rpm. Samples were collected every 5 min for 120 min and phage titers were determined by the double-layer agar technique. Then, the latent period and burst size were calculated [28].

### 4.8. Inactivation of Aeromonas hydrophila N17 in Vitro

The method used in this study was adapted from Jun et al. [12] and Le et al. [23] with some modifications described below. *A. hydrophila* N17 was streaked onto sheep blood agar (OXOID, UK), incubated at 30 °C overnight, and harvested on LB to have a final concentration of 10^7^ CFU/mL. A 10 mL suspension of the *Aeromonas* sp. in LB (around 10^7^ CFU/mL) was then mixed with same volume of a phage preparation (concentration of 10^5^ to 10^7^ PFU/mL) to reach multiplicity of infection (MOI) of 0.01, 0.1, 1 (http://www.bio-protocol.org/e1295). A 20 mL sample of *Aeromonas* sp. in LB (~×10^7^ CFU/mL) was used as a control. The mixture was incubated at 30 °C and 150 rpm. Samples were taken every 30 min for 8 h to determine the exact time of the appearance of phage-resistant bacteria, and every 60 min for the next 6 h to determine the increase in the concentration of phage-resistant bacteria. Then, samples were withdrawn every 3 h to the end of the experiment. The concentration of the *Aeromonas* sp. was measured by optical density determination at 550 nm using a spectrophotometer (Thermo Scientific Genesys 20, Waltham, MA, USA).

### 4.9. Phage Treatment of Infected Fish

A total of 360 healthy catfish (*Pangasianodon hypophthalmus*) (30 g/fish) were divided into 12 groups in 50 L plastic tanks at 30 ± 1 °C. All treatment fishes were infected intraperitoneally with *A. hydrophila* N17 (final concentration: 3.2 × 10^6^ CFU/fish) and were then immediately injected with a phage cocktail (MOI 0.01, 1 and 100). A positive control was composed of fishes injected with *A. hydrophila* N17 only. Negative controls 1 and 2 were fishes with no injection and fishes injected with fluid separated from the broth containing bacteria and medium, respectively. The mixed phage preparation consisted of Φ2 and Φ5.

The mortality rates of the fishes were recorded every 12 h for eight days, and the kidneys of both the dead and surviving fishes were subjected to a bacterial isolation study [3]. Bacteria isolation was carried out from all dead fishes, indicating that the deaths were caused by *A. hydrophila* [3]. All treatments were performed in duplicates.

The animal experiment was conducted according to the animal ethical guidelines of the Vietnamese government (project supported by Vietnam Ministry of Agriculture and Rural Development, 2016–2018, number: 04/TCTS-KHCN-HTQT-DT 2016).

### 4.10. Statistical analysis

IBM SPSS Statistics 20 software was used to analyze the data. Single factor ANOVA was applied to test for differences in the fish numbers in the *Aeromonas*-infected fishes receiving or not the phage therapy (*p* < 0.05). Standard deviations were calculated in all experiments. 

## 5. Conclusions

The phages Φ2 and Φ5, belonging to the *Myoviridae* family, were successfully isolated and displayed inhibition of the growth of the *A. hydrophila* strains tested. The results obtained from the use of a phage cocktail indicate that phages can be used successfully for the treatment of *Aeromonas* infections in catfish via intraperitoneal injection. Phages may therefore be considered as potential biocontrol agents to combat *Aeromonas* infections in fish farms.

## Figures and Tables

**Table 1:** Antibiogram profile of the *Aeromonas hydrophila* strains tested.

Antibiotics	Number of Resistant Isolates (*n* = 6)
Tetracycline	2
Oxytetracycline	6
Gentamycin	6
Kanamycin	5
Bactrim (SMX/TMP)	6
Doxycycline	2
Enrofloxacin	6
Amoxicillin/clavulanic acid	6
Ampicillin	6
Ciprofloxacin	2

**Table 2:** Characteristics of bacteriophages against *A. hydrophila* strains.

Φ	Concentration PFU/mL	Head (nm)		Neck (nm)		Tail Sheath (nm)		Genus
		L	W	L	W	L	W	
2	10^9^		129	10	15	173	15	*Spounalikevirus*
5	10^10^		120	15	15	198	15	*Spounalikevirus*

W: width; L: length.

## Supplementary Materials

The following are available online at http://www.mdpi.com/2079-6382/7/1/16/s1, Table S1: The OD_550nm_ value of *A. hydrophila* N17 with the effect of Mitomycin C over the incubation time at 30 °C; Table S2: Host range of the phages against *Aeromonas* species.
