# Supplementary material for: Protective Effects of Bacteriophages against Aeromonas hydrophila Causing Motile Aeromonas Septicemia (MAS) in Striped Catfish
Source: Antibiotics (Basel). 2018 Feb 25;7(1):16. doi: 10.3390/antibiotics7010016 (PMC5872127; doi:10.3390/antibiotics7010016)
Supplement: Supplementary file 1 [file antibiotics-07-00016-s001.pdf]

antibiotics

Supplementary Material

Protective Effects of Bacteriophages against *Aeromonas hydrophila* Species Causing Motile Aeromonas Septicemia (MAS) in Striped Catfish

Tuan Son Le, Hien Nguyen Thi, Phuong Vo Hong, Cuong Doan Van, Loc Nguyen Hong, Trung Cao Thanh, Tuan Tran Trong, Paul C. Southgate and D. İpek Kurtböke

Table S1. Growth of *A. hydrophila* N17 under the effect of Mitomycin C.

Mitomycin C concentration	OD_{550nm}						
	**0 h**	**1 h**	**2 h**	**3 h**	**4 h**	**5 h**	**6 h**
0 ug/mL (Control)	0.200 ± 0.081	0.948 ± 0.011	2.566 ± 0.082	4.295 ± 0.658	5.855 ± 0.997	6.210 ± 0.651	5.560 ± 0.184
0.1 ug/mL		0.957 ± 0.023	2.488 ± 0.034	4.625 ± 0.290	5.440 ± 0.283	5.355 ± 0.318	5.430 ± 0.368
0.5 ug/mL		0.933 ± 0.004	2.038 ± 0.122	2.585 ± 0.092	2.295 ± 0.120	1.755 ± 0.290	1.224 ± 0.122

Table S2. Host range of the phages against *Aeromonas* species.

Bacteria	Strain codes	Isolation location *	Date of collection	Gene	LD_{50} (CFU/mL)	Φ2	Φ5
A. hydrophila strains	Ah 01	VL	24/06/2014	g14		+	+
	Ah 02	VL	03/10/2012	g14		+	+
	Ah 03	VL	03/10/2012	g14		+	+
	Ah 04	CT	12/01/2013	g14		+	+
	Ah 08	DT	14/01/2013	g14		+	+
	Ah 13	BT	02/02/2013	g11		+	+
	B122	CT	03/10/2012	g14		+	+
	B126	AG	16/08/2012	g10		+	+
	B167	BT	02/02/2013	g14		+	+
	B173	BT	02/02/2013	g14		+	+
	B44	VL	02/10/2012	g14		+	+
	B60	VL	03/10/2012	g14	3.3×10^2	+	+
	B61	VL	03/10/2012	g14		+	+
	B64	VL	03/10/2012	g14		+	+
	B65	VL	03/10/2012	g14		+	+
	B66	VL	03/10/2012	g14		+	+
	N17	AG	12/11/2012	g14	5.7×10^4	PH	PH
	ATCC 35654					+	+
Aeromonas spp. (23).	Ac11	VL	25/09/2012	g03		-	-
	Av12	DT	14/01/2013	g01		-	-
	B101	CT	12/11/2012	g01		-	-
	B105					-	-
	B110	DT	11/01/2013	g02		-	-
	B12	AG	23/08/2012	g13		-	-
	B168	BT	02/02/2013	g03		-	-
	B174	BT	02/02/2013	g01		-	-
	B45	VL	02/10/2012	g02		-	-
	B58	VL	03/10/2012	g13		-	-
	B71	CT	24/09/2012	g08		-	-
	B85	DT	30/10/2012	g09		-	-
	B87	DT	30/10/2012	g13		-	-
	B88	DT	30/10/2012	g05		-	-

Bacteria	Strain codes	Isolation location *	Date of collection	Gene	LD50 (CFU/mL)	Φ2	Φ5
	CL73	TG	06/08/2014	g13		-	-
	K13	DT	11/10/2012	g14		-	-
	K7	DT	11/10/2012	g14		-	-
	N1	AG	15/08/2012	g13		-	-
	N15	VL	13/10/2012	g13		-	-
	N19	DT	11/01/2013	g02		-	-
	N21	DT	11/01/2013	g01		-	-
	N4	AG	16/08/2012	g09		-	-
	N40	DT	14/01/2013	g09		-	-
	N6	AG	16/08/2012	g10		-	-
	A. caviae (ATCC 15468)					-	-
	A. salmonicida (ATCC 33658)					-	-
	A. salmonicida (ATCC 7965)					-	-
Edwardsiella tarda	ATCC 15947					-	-
Edwardsiella ichtaluri	LMG 7860					-	-
Hafnia alvei	ATCC 51815					-	-
Streptococcus agalactiae	ATCC 12386					-	-
Vibrio parahaemolyticus	ATCC 17802					-	-

"+": susceptible to phage lysis; "-": not susceptible to phage lysis; * Locations in Vietnam: VL: Vinh Long; CT: Can Tho; DT: Dong Thap; BT: Ben Tre; AG: An Giang.; PH: propagation host; g01: no toxic gene; g02: *alt*, g03: *alt-ahp*A; g04: *ahh*1-*alt*, g05: *ahh*1-*alt-lip*; g06: *ahh*1-*alt-ahp*A; g07: *ahh*1-*alt-ahp*A-*ast*; g08: *ahh*1-*alt-ahp*A-*lip*; g09: *aer*A-*ahh*1-*alt*; g10: *aer*A-*ahh*1-*alt-lip*; g11: *aer*A-*ahh*1-*alt-ast*; g12: *aer*A-*ahh*1-*alt-ahp*A; g13: *aer*A-*ahh*1-*alt-ahp*A-*lip*; g14: *aer*A-*ahh*1-*alt-ahp*A-*lip-ast*.
